# Time to Evolve Beyond Prototypical Community-Based Distribution (CBD) of Contraception?

**DOI:** 10.9745/GHSP-D-18-00462

**Published:** 2018-12-27

## Abstract

CBD efforts have a definite role in a variety of country programming contexts. However, contemporary efforts need to strive for an expanded method mix, strong support and motivation of CBD agents, and robust integration with existing health systems.

See related article by Hernandez.

In this issue of GHSP, Hernandez and colleagues present a notable description of how their process evaluation of a large community-based distribution (CBD) program in the capital city of the Democratic Republic of the Congo (DRC), Kinshasa, led to major improvements in that program.^1^ We compliment the authors for forthrightly detailing the weaknesses they observed in the original design of the AcQual program and the reasonable changes they subsequently have made. Nevertheless, the experience offers the opportunity to ask what might be the proper role for similar CBD efforts in the future.

Community-based distribution of contraception has had a number of variations, including:

- Whether agents are volunteers or paid (or retain proceeds from sales of the contraceptives),
- Which contraceptive methods they provide,
- Whether agents are expected to actually visit clients in their homes, and
- Whether CBD programs should focus just on contraception or include other health interventions.

Historically, CBD programming was pivotal, especially in the 1970s and 1980s, in demonstrating that a substantial proportion of women would use contraception if it were readily available, getting contraception to some extent out of the “medical model” and helping to jump-start and advance family planning efforts in early programs. Yet since then, many countries have made marked progress socioeconomically and have made major improvements in their health programs. Moreover, more contraceptive options, notably the popular implants and injectables (including subcutaneous DMPA), are much more widely available, and other successful service delivery modes—notably mobile outreach service delivery; social marketing in drug shops, pharmacies, and community outlets; and social franchising of private health clinics—have evolved to reach geographically dispersed clients.

But importantly the overall global health agenda is vastly larger going forward. Accordingly, the much broader concept of “community health worker” in its many variants and with a correspondingly broader health intervention mandate has taken root and occupies a key place in many low- and middle-income country programs. A good reference on best practices for community health workers for family planning is the High Impact Practice brief.^2^

The original CBD model undertaken by Hernandez et al. was “prototypical” in some ways. It focused only on contraception, relied on volunteer CBD agents (albeit they retained some of the proceeds of contraceptive sales), had only a very limited set of methods that the agents could provide (male condoms, oral contraceptives, and CycleBeads for the Standard Days Method), and was funded by external donors. Simply getting family planning moving more in the DRC is laudable. And to be sure, the program designers faced some constraints and made accommodations toward synchrony with the ongoing public-sector system. They had policy limitations as to what methods the agents could provide. They used existing contraceptive supply chain and service delivery records. And they linked the CBD agents with existing clinical family planning services.

As described by Hernandez et al., it is fairly evident, however, that in its original form, the project was far from successful. They describe a variety of shortcomings, to a large extent related to the challenging environment and systems they worked in. Thus, total annual couple-years of protection (CYPs) provided by the CBD agents were only roughly 36,000. Consider the contrast with other successful family planning efforts in the DRC:

- Social marketing efforts in the DRC registered 1.95 *million* CYPs in 2017^3^
- Successful provision of injectables by nursing students^4^
- Highly successful provision of long-acting reversible methods, especially implants^5^
- PMA2020 surveys in Kinshasa showing dominant and growing use of injectables and implants,^6^ documenting that substantial progress is being made through other service delivery channels

Changes proposed for the upcoming iteration of the program—AcQual 3—appear reasonable, especially in terms of allowing the CBD agents to provide subcutaneous DMPA. And the AcQual experience seems likely to have contributed to the adoption of such progressive family planning policies in the DRC. The extent to which AcQual 3 will be more successful in this challenging environment remains to be seen. It should be recognized, however, that implementation will involve considerable effort, and interaction with other health programming and the long-term sustainability within the government system is unclear.

It is axiomatic in family planning programming that (1) when more contraceptive methods are available and (2) clients have more programmatic modalities from which to choose, more clients' needs will be satisfied and overall programming will be more successful. Still, undertaking any additional service delivery modality entails considerable effort and cost. And alignment with other health interventions and consideration of long-term sustainability is key. Accordingly, consideration of if, where, when, and how to embark on a new family planning CBD effort calls for careful and considerable thought and selectivity. *–Global Health: Science and Practice*
